# Evaluation of Feature Extraction and Recognition for Activity Monitoring and Fall Detection Based on Wearable sEMG Sensors

**DOI:** 10.3390/s17061229

**Published:** 2017-05-27

**Authors:** Xugang Xi, Minyan Tang, Seyed M. Miran, Zhizeng Luo

**Affiliations:** 1 School of Automation, Hangzhou Dianzi University, Hangzhou 310018, China; 11045404@hdu.edu.cn (M.T.); luo@hdu.edu.cn (Z.L.); 2 Department of Mechanical Engineering, University of Akron, Akron, OH 44325, USA; sm201@zips.uakron.edu

**Keywords:** surface electromyography (sEMG), feature extraction, classifier, activity monitoring, fall detection

## Abstract

As an essential subfield of context awareness, activity awareness, especially daily activity monitoring and fall detection, plays a significant role for elderly or frail people who need assistance in their daily activities. This study investigates the feature extraction and pattern recognition of surface electromyography (sEMG), with the purpose of determining the best features and classifiers of sEMG for daily living activities monitoring and fall detection. This is done by a serial of experiments. In the experiments, four channels of sEMG signal from wireless, wearable sensors located on lower limbs are recorded from three subjects while they perform seven activities of daily living (ADL). A simulated trip fall scenario is also considered with a custom-made device attached to the ankle. With this experimental setting, 15 feature extraction methods of sEMG, including time, frequency, time/frequency domain and entropy, are analyzed based on class separability and calculation complexity, and five classification methods, each with 15 features, are estimated with respect to the accuracy rate of recognition and calculation complexity for activity monitoring and fall detection. It is shown that a high accuracy rate of recognition and a minimal calculation time for daily activity monitoring and fall detection can be achieved in the current experimental setting. Specifically, the Wilson Amplitude (WAMP) feature performs the best, and the classifier Gaussian Kernel Support Vector Machine (GK-SVM) with Permutation Entropy (PE) or WAMP results in the highest accuracy for activity monitoring with recognition rates of 97.35% and 96.43%. For fall detection, the classifier Fuzzy Min-Max Neural Network (FMMNN) has the best sensitivity and specificity at the cost of the longest calculation time, while the classifier Gaussian Kernel Fisher Linear Discriminant Analysis (GK-FDA) with the feature WAMP guarantees a high sensitivity (98.70%) and specificity (98.59%) with a short calculation time (65.586 ms), making it a possible choice for pre-impact fall detection. The thorough quantitative comparison of the features and classifiers in this study supports the feasibility of a wireless, wearable sEMG sensor system for automatic activity monitoring and fall detection.

## 1. Introduction

As a result of an aging population, the number of elderly or frail people who need help in their daily activities is rapidly increasing [1,2,3]. This leads to a series of problems in caring for older people and people with medical disabilities. Falls are the leading cause of trauma and death among people 65 or older and the resulting health care costs represent a serious public burden [1]. Helping this group of people to have a better life has many social benefits. With the development of the wireless network and wearable sensing technology, wireless, wearable and reliable sensors provide the realization of context awareness in wireless body area network (WBAN). The wearable sensors provide a central device called the coordinator with raw data collected from human body. The coordinator processes the data, and the result can then be used for diagnostics, prescription, wellness monitoring, entertainment, rehabilitation, or security purposes [2]. One essential basis for context-aware applications is the daily activity monitoring, which monitors a user’s biological signals, movement patterns, or body posture [2], and further does activity recognition [3,4]. The capability of automatically identifying the activities could improve the objectivity and comprehensiveness of a patient’s physical performance record and further offers intelligent assistance for patients suffering from cognitive disorders such as Parkinson’s or Alzheimer’s diseases [3]. The other important applications are fall detection [5,6] and rehabilitation [7].

Compared with vision or acoustic sensors, wearable sensors provide important advantages such as their compatibility with diverse physical environments, the feasibility of outdoor operations, and no disclosure of privacy. One major concern for wearable sensors is the user’s compliance with wearing additional devices. However, with wearable sensors becoming smaller, lighter, and wireless, they can be worn in band- and ring-like fashion, or even embedded in clothes, causing little obstruction when employed for activity awareness applications [4,8,9,10], and hence making them promising in a wide range of uses, such as those related to walking issues, paralysis, neurological disorders and even walking aids. Recent systems use gyroscopes, acceleration, heart rate monitors, EMG and other kinds of sensors to help building a wearable device.

Due to their relative ease of use and versatility, accelerometer (ACC) or gyroscope sensors are the most common sensors used in activity monitoring [11] and fall detection [12]. ACC sensors collect the information of both the acceleration and the angle between gravity and the acceleration. The acceleration energy can be distinguished between static and dynamic activities. Thus, a threshold can be set for ADLs-detection with the use of accelerometer [12]. Existing studies can guarantee an average recognition rate about 90% using SVM, fuzzy neural networks, etc., [13,14,15]. 

Despite their long-standing dominant role, ACC sensors have shortcomings. For example, ACC sensors can hardly distinguish between active versus passive movements, meaning that these sensor do not work well for users in elevators, cars, or subways. They also have difficulty in detecting abnormal movements, like a mechanical tremor or vibration. These sensors are not good at differentiating between loaded and non-loaded condition, e.g., stair-ascending carrying no load versus a 3 kg sandbag. Last but not least, ACC sensors can hardly detect static activities, e.g., sitting in the chair for several hours could possibly not be distinguished from exercising on a stationary bicycle with fixed-position handlebars [3].

To overcome the aforementioned problems of ACC sensors, some studies have tried to use an additional gyro to detect the postures at much higher cost [16]. As another widely used sensor in monitoring activities, gyros detect the movement of the human body by the transformation of triaxial and geomagnetic triangular angles. For example, Nyan used three gyroscope sensors at three different locations, and explored sideways and backward falls from normal activities of daily living using angular rate sensors (gyroscopes). Despite the 100% sensitivity, 16% of ADL events tested were misinterpreted as falls [17]. The main drawback of gyro is its drift caused by disturbed torque, which would generate false angular velocity.

In contrast with accelerometers or gyroscope sensors, surface electromyography (sEMG) sensors which measure the electrical potentials generated by muscle activity using noninvasive electrodes placed on the skin surface, have an inherent advantage in distinguishing passive and active activities, predicting movements and getting a short calculating time, thus making it a possible solution to overcome the above difficulties caused by accelerometer and gyroscope sensors.

EMG is not yet a popular choice of detecting activities, for the following reasons: first, the quality of EMG data can be affected by movement artifacts, physical contact/pressure, sweat and muscle fatigue [18]. Besides, EMG signals are weak, thus adding to the difficulty in processing the raw data. A second concern is that the quality of EMG signals has a high relationship with the sensor location [7]. However, first, with the fast development of sensor technology, many sensors are designed with amplifiers, software selectable filters and motion artifact suppression, like the Trigno™ Wireless EMG System and SX230FW of Biometrics. Secondly, many studies have considered the knowledge of anatomical landmarks for the location of EMG sensors [1,3,7,19,20,21,22,23,24,25,26], and indeed recent years have seen the fast development of EMG technology in monitoring wearable systems. 

Studies have found that sEMG signals could be successfully applied to gesture recognition, gait analysis, limb prosthetic control, etc. [20,21,22]. An EMG sensor is employed in [27] to detect the muscle activities during human locomotion and captures the human walking dynamics for motion recognition and step detection in a Pedestrian Dead Reckoning (PDR) solution. Myers studied the feasibility of AgNWs electrodes for measuring sEMG signals, which were flexible, wearable, and potentially robust for daily use [28]. Recent developments in EMG technology have increased the feasibility of implementation of an EMG-based monitoring system appropriate for use in the home or community. These developments include portability, in the form of data loggers and telemetry systems, and the miniaturization of sensors, which include on-board circuitry to minimize the physical size of the system and enhance patient usability. EMG signals have inherent attributes that make them ideally suitable for monitoring functional activities. They provide information from the different muscle groups that are active during a motor activity; hence they have the potential to uniquely identify the different activities [23].

With respect to fall detection, it is desirable to develop a system capable of detecting falls in advance of an injury. This is difficult because of the low lead-time before the impact (less than 700 ms). Compared with inertial sensors like ACC or gyro sensors, EMG has a decisive factor in this aspect. Laboratory data show that inertial sensors detect a fall-action in 400 ms [29,30], while in order to realize pre-impact fall detection [1], the detection time must be less than 300 ms [31]. EMG sensors would reduce detection time to 200 ms or less [29]. Furthermore, in contrast with accelerometer or gyroscope sensors, sEMG can recognize the movement in advance [4,32]. For this reason, EMG-based solution has drawn much attention for fall detection. 

EMG signal activation is associated with the muscle contraction and can be used to identify the motion. Researchers have been working on this issue for several decades [1,3,8,9,10,19,20,21,22,23,24,25,26]. The critical problem of these investigations is the choice and computation of effective features from the signals and classification techniques. Feature extraction is generally divided into the time domain, frequency domain, time-frequency domain, and entropy-spectrum. Among these, the time domain analysis is the most common method because it is computed based on the signals’ amplitude, which can be easily calculated. Arief et al. [33] analyzed Mean Absolute Value (MAV), Variance (VAR), Willison Amplitude (WAMP), Waveform Length (WL), and Zero Crossing (ZC) from the time domain, aiming to find the best way to minimize the complexity of implementation and reduce the cost of information processing. For the frequency domain, two modified mean and median frequencies are presented for robust feature extraction [34]. Time-frequency domain, a combination of time and frequency, can characterize varying frequency information at different time locations, providing plenty of non-stationary information about the analyzed signals [35]. One of the methods, Wavelet Transform (WT), a research hotspot in recent decades, is more appropriate for representing short bursts of high-frequency signals or long-duration, slow-varying signals [36]. The Wavelet Packet Transform (WPT), a crucial extension of WT, can effectively eliminate the high frequency noises and extract the characteristics of EMG signals [37]. However, the WT is still poor in high-frequency band and WPT lacks a translation-invariant property. Therefore, a new solution was proposed to use the wavelet packet node energy to construct the feature vector [38]. Entropy-spectrum, as a measure of disorder or uncertainty in the data, was introduced by Shannon. To date, many different types of entropy methods have been used in many diverse applications within the biomedical domain [39]. Diab et al. [40] investigated the performance of the sample entropy, which was applied to real uterine EMG signals to distinguish between pregnancy and labor contraction bursts.

Another important step in activity monitoring and fall detection is the classification technique selection. For systems with a few inputs, the most common algorithm for classification, especially for the statistical feature evaluation and classification, is the Linear Discriminant Analysis (LDA). Though accurate and fast, its use becomes complicated for multi-input and multi-output systems. To address this problem, the so-called “kernel-trick” was taken into account. For example, Nonparametric Weighted Feature Extraction (NWFE), Principal Component Analysis (PCA), kernel PCA with Gaussian kernel, and kernel PCA with polynomial kernel were suggested for classification [38]. Kakoty et al. [36] used a linear kernel Support Vector Machine (SVM) with discrete wavelet transform to classify six grasp types, which showed a recognition rate of 84 ± 2.4%. Based on machine learning theory, the SVM is the state-of-the-art classification method, which has significant advantages due to its high accuracy, elegant mathematical tractability, direct geometric interpretation, and lack of a need for a large number of training samples to avoid overfitting [41]. To achieve a higher efficiency, Fuzzy Min-Max Neural Network (FMMNN), whose learning phase is single-pass-through and online-adaptive, was studied. This also led to other modified methods like multi-level fuzzy min-max (MLF) classifier, which mainly uses a multi-level tree structure handles the overlapping area problem [42]. Other widely used unsupervised learning methods are clustering techniques. Fuzzy c-means (FCMs) data clustering was used to automate the construction of a simple amplitude-driven inference rule base, which resulted in the overall classification rates of lower-limb actions ranging from 94% to 99% [43].

In retrospect, a few studies can be found for the quantitative performance comparison of feature extraction and classification of sEMG in the context of controlling prosthetic limbs or gait phase recognition [44,45], but almost no studies can be found for the quantitative performance comparison of activity monitoring and fall detection. For systems with a good performance, EMG features should be selected in maximum class separability, high recognition accuracy and minimum computational complexity, ensuring as low as possible misclassification rate in real-time implementation with reasonable hardware [44]. The current research is aimed at selecting the best sEMG features and classification method from the three approaches mentioned above for the recognition of daily activities and falls.

The remainder of this paper is structured as follows: Section 2 outlines daily activities and falls, and data acquisition. Section 3 presents various feature extraction techniques and classification methods. The analysis of experiments performed are described in Section 4. The conclusions and discussions are presented in Section 5 and Section 6, respectively.

## 2. Activity Monitoring and Data Acquisition

In order to achieve daily activity monitoring and fall detection, it is necessary to distinguish daily activities and falls. The most common three activities of daily living (ADL) were selected, i.e., walking, stair-ascending and stair-descending. Four ADLs, stand-to-squat, squat-to-stand, stand-to-sit, and sit-to-stand, were selected as well. They are not easily distinguished from falling or each other.

**Figure 1:**
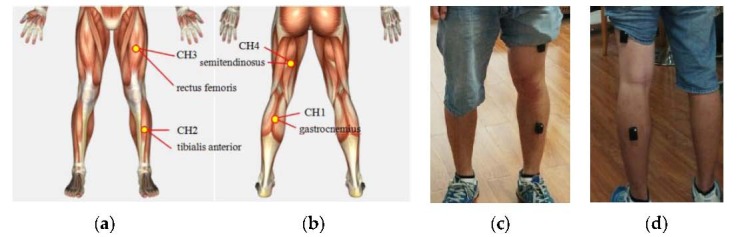
Muscles channels and sEMG sensors placement. (**a**) Forward muscle; (**b**) Backward muscle; (**c**) Forward sensors placement; (**d**) Backward sensors placement.

Since the activities mentioned above result from contraction of the muscles in the lower limbs, four surface electrodes were used to measure sEMG signals from gastrocnemius, rectus femoris, tibialis anterior, and semitendinosus, which are muscles with lower limb motions. The sEMG electrodes were placed upon muscles of the left lower limb, indicated by small circles in Figure 1 marked by CH1 through CH4. Semitendinosus plays a crucial role in stretching the hips, flexing the legs and rotating the knee joints externally [46]. Gastrocnemius is mainly concerned with standing and walking activities. Rectus femoris is a powerful knee extensor, which has a role in flexing the hips, and the tibialis anterior muscle’s roles are mainly regarding stretching the ankle and enabling the foot eversion [47]. 

The sEMG signal was recorded using Trigno™ Wireless EMG (Delsys Inc, Natick, MA, USA), which provided a 16-bit resolution, a bandwidth of 20–450 Hz, and a baseline noise <1.25 μV (rms). It has a typical operating range of 40 m and the communication protocol is Bluetooth. It has a motion artifact suppression (patent) that can be freely moved. The sEMG signals were sampled at 1024 Hz using EMGworks 4.0 acquisition software (DelSys Inc.). All sensors were secured to the skin by a double-sided adhesive interface. A reference electrode was attached to the skin near the SEMG electrodes to supply a voltage baseline.

## 3. Algorithm Description

### 3.1. Feature Extraction

Surface EMG features were computed using 1.5 s epochs (1536 samples), which was the time necessary to complete the longest activity (stand-to-squat) in our experiment. And we collected data for each activity separately and get the features.

For the purpose of comparison, 15 well-known EMG feature types were considered as shown in Table 1, where N and *x_i_* denote the number of samples and the *i*-th raw EMG sample, respectively, and *u*(*x*) indicates a unit-step function.

(1) Integral of Absolute Value (IAV)

In case of discrete signals, IAV is represented as the average of the absolute value of each signal sample, and the formula is as follows [4]:

(1) $$IAV=\frac{1}{N}\sum_{i=1}^{N}\left|x_{i}\right|$$

(2) Variance (VAR)

In the stochastic process, variance characterizes the average power of a random signal and can be explained as follows [4]:

(2) $$VAR=\frac{1}{N-1}\sum_{i=1}^{N}x_{i}^{2}$$

(3) Wilson Amplitude (WAMP)

This is the number of times that the difference between two consecutive amplitudes exceeds a certain threshold. It can be formulated as:

(3) $$WAMP=\sum_{i=1}^{N}u(\left|x_{i+1}-x_{i}\right|-T)$$

In this study, a threshold *T* of 0.05 V is considered. This feature is an indicator of firing motor unit action potentials (MUAP) and therefore an indicator of the muscle contraction level [25].

(4) Zero Crossing (ZC)

ZC represents the number of times that the amplitude of the signal passes through zero [48]:

(4) $$ZC=\sum_{i=1}^{N-1}u(-x_{i}x_{i+1})$$

(5) Number of Turns (NT)

NT counts the number of changes in the sign of the slope, in other words, the number of signal peaks [49]:

(5) $$NT=\sum_{i=1}^{N-2}u[(x_{i+1}-x_{i})(x_{i+1}-x_{i+2})]$$

(6) Mean of Amplitude (MA)

This feature determines the mean of the difference in amplitudes of two consecutive samples [44]:

(6) $$MA=\sum_{i=1}^{N-1}\left|x_{i+1}-x_{i}\right|$$

(7) Mean Frequency (MF)

This feature estimates the mean frequency of the signal in a time segment [50]:

(7) $$MF=\frac{\sum_{i=1}^{N}h_{i}f_{i}}{\sum_{i=1}^{N}h_{i}}$$

where *f_i_* denotes frequency, and *h_i_* denotes intensity of frequency spectrum.

(8) Histogram (HIST)

HIST contains a series of highly unequal vertical stripes or segments representing data distribution [49]. This study considers the amplitude range as −5 V–5 V and then divides it into 21 amplitude slots of equal size.

(9) Auto-Regressive Coefficient (AR)

In the Auto-Regressive model, the signal samples are estimated by the linear combination of their earlier samples. This process computes linear regression coefficients. It has been shown that the EMG spectrum changes with muscle contraction that results in change in AR coefficients [51]. Various experimental and theoretical studies have shown that the model order P = 4 is suitable for EMG signals [52]. Therefore, it was used in the current research. 

(10) Auto-Regressive Coefficient From Third-Order Cumulant (ARCU) 

The ARCU is the AR from the third-order cumulant of the signal in each time segment. The novel part of this method is that the input of the algorithm is the cumulant rather than an auto-correlation function. Normally ARCU can effectively separate recycle stationary signals and stationary signals, and completely suppress Gaussian colored noise in theory. Here, a fourth-order AR model from the third-order cumulant is used [44].

(11) Energy of Wavelet Coefficient (EWT)

This feature computes the energy of the wavelet-transformed signal:

(8) $$F_{j}=\sqrt{\frac{1}{K}\sum_{k=1}^{K}W_{j,k}^{2}}$$

where *F_j_* is the coefficient of wavelet energy. *K* is the number of the *j*-th layer decomposed coefficient. *W_j,k_* is the *k*-th coefficient of the *j*-th layer decomposed coefficient. Db8 wavelet and decomposition layer 5 is used in our study. 

(12) Energy of Wavelet Packet Coefficient (EWP)

This feature computes the energy of the wavelet packet transformed signal. It is similar to the EWT. Compared with EWT, the advantage of EWP is that it can deal with both high and low frequency components, but the number of feature components is increased, therefore the computation complexity is also increased [34].

(13) Zero Crossing of Wavelet Coefficient (ZCWT)

It is a similar algorithm to ZC. It calculates the number of base-line crossings in the wavelet domain [53]

(9) $$ZCWT=\sum_{j=1}^{K}u(-W_{j}W_{j+1})$$

where *W_j_* is *j*-th layer decomposed coefficient.

(14) Fuzzy entropy (FE)

Fuzzy entropy describes the degree of fuzziness of fuzzy sets, which used to quantify the regularity of time series. The formula is as follows:

(10) $$FuzzyEn(N,m,r)=\underset{N\to +\infty }{\lim}(\ln\phi_{m}-\phi_{m+1})$$

(11) $$\phi_{m}=1/(N-m)\sum_{i=1}^{N-m}\left[1/(N-m-1)\right]\sum_{j=1,j\neq i}^{N-m}D_{ij}^{m}$$

where *N* is the number of samples, *m* defines the dimension of the data, *D_ij_* is the similarity degree of two samples, *r* is the width of the exponential function in *D_ij_*, and $\phi_{m}$ is called the mean average similarity [54].

(15) Permutation entropy (PE)

The permutation entropy is a way of quantifying the relative occurrence of the different motifs [55], which is based on the complexity of the measurement, applies to non-linear signal, and has a high anti-interference ability and a good robustness. The core of PE is choosing n consecutive points of samples and making up an n-dimensional vector. The obtained signals are sorted in ascending order. The permutations and combinations of the new sequence is one of n!. Then probability statistics of various permutations and combinations in the entire time series is calculated. It is symbolized as p(π), in which *π* represents different permutations ways [56]. The formula is as follows:

(12) $$H(n)=-\sum_{\pi =1}^{n!}p(\pi )\ln(p(\pi ))$$

### 3.2. Feature Class Separability

In order to perform a qualitative evaluation of the extracted feature, the Fisher’s discriminant function was used to translate data samples into a class separability index. 

To achieve the class separability index, the trace of the between-class scatter matrix is divided into the trace of the within-class scatter matrix [57]. 

The between-class scatter matrix *S_B_* is defined as follows:

(13) $$S_{B}=\sum_{i=1}^{k}(m_{i}-m_{m}){\left(m_{i}-m_{m}\right)}^{T}$$

*S_B_* is the covariance matrices of the means of all classes in which *m_m_* is the mean of all the classes’ means and *m_i_* is the mean of the *i*-th class.

The within-class scatter matrix *S_w_* is defined as follows:

(14) $$S_{w}=\sum_{i=1}^{k}E((x-m_{i}){\left(x-m_{i}\right)}^{T})$$

*S_w_* is the mean of the covariance matrices of all classes in which *m_i_* is the mean of the *i*-th class and *x* is the sample vector.

The class separability index is calculated as:

(15) $$J=\frac{\mathrm{trace}(S_{b})}{\mathrm{trace}(S_{w})}$$

It is obvious that the quality of the space feature will improve when the value of the index increases.

### 3.3. Classification

Five representative classification techniques (shown in Table 2) were considered and listed below:

(1) Fisher Linear Discriminant Analysis (FDA)

The FDA, also known as Fisher’s Linear Discriminant Analysis (LDA), finds a linear combination of features that characterizes or separates two or more classes of objects or events. The resulting combination may be used as a linear classifier or for dimensionality reduction before later classification [57].

(2) Fuzzy Min-Max Neural Network (FMMNN) 

The FMMNN is based on the hyperbox fuzzy sets. A hyperbox is defined by its minimum and maximum points which are created by the input patterns [58]. The membership function is set with respect to the minimum and maximum points of the hyperbox [59]. Its multilayer structure is capable of dealing with a nonlinear separability issue. It also possesses an adaptive learning capability.

(3) Gaussian Kernel Fisher Linear Discriminant Analysis (GK-FDA)

Kernel Fisher Linear Discriminant Analysis (KFDA) is the evolution of the FDA and it calculates the projection by kernel function rather than Fisher’s algorithm. In a real experiment, most of the kernel methods solve a linear problem in the kernel feature space [60]. In the current study, Gaussian kernel, the most pervasive kernel, is used.

(4) Gaussian Kernel Support Vector Machine (GK-SVM)

It is a nonlinear version of the SVM classification. Kernel trick with SVM is the most used kernel classifier among the available kernel methods. It makes the SVM more robust and flexible for any kind of data irrespective of its linearity to achieve a highly accurate classification rate [60]. 

(5) Fuzzy C-means algorithms (FCM)

Fuzzy C-means (FCM) is a method of clustering that allows data to belong to two or more clusters [61]. Fuzzy C-means model aims to get membership degree of each sample point in all classes through optimization of the objective function. This function determines the sample type and fulfills the purpose of automatic sample data classification. The common Fuzzy C-means model is an unsupervised machine learning that analyzes and models data with fuzzy theory. 

## 4. Experiments and Results 

**Figure 2:**
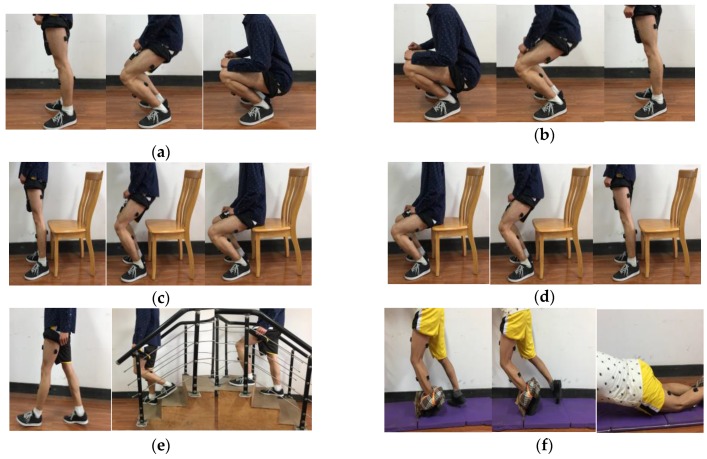
Seven activities of daily living and trip-fall in the experiment. (**a**) stand-to-squat; (**b**) squat-to-stand; (**c**) stand-to-sit ; (**d**) sit-to-stand; (**e**) walking, stair-ascending and stair-descending; (**f**) trip-fall.

Three subjects (two males and one female, age 24–26, height 160–180 cm, weight 48–70 kg) without neural or musculoskeletal deficits were randomly recruited for the experiment. Each subject performed seven activities of daily living (ADLs): stand-to-squat, squat-to-stand, stand-to-sit, sit-to-stand, stair-ascending, stair-descending, and walking. In addition, a few unexpected simulated trip falls induced by a custom-made device attached to the ankle, were interspersed among the normal walking trials. The custom-made device attached to the ankle is made by a round cushion and a rope. The participants repeated the procedure for 10 times in each experiment day, making sure that the total times of each activity and trip fall was at least 30, and the order of activities stayed same for each experiment. The experiment scene is shown in Figure 2.

**Figure 3:**
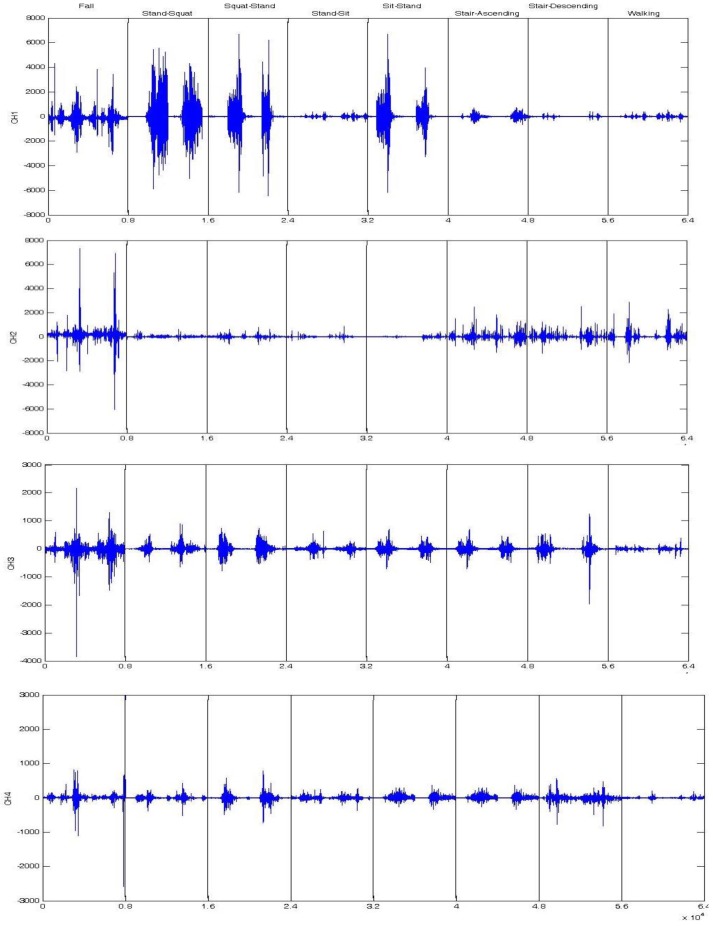
Examples of raw sEMG signals of some typical activities.

Typical EMG signals recorded from a typical subject are shown in Figure 3, illustrating the raw sEMG signals of eight typical activities used in this paper. The sEMG signals burst only at the posture transition. During the period of the posture transition, sEMG signals have obvious ups and downs, and the magnitude of some of the transition roses up to 7 mV. The trip falls have a relatively obvious change in most channels. Squat-to-sit and sit-to-squat had a similar EMG with a high magnitude in Channel 1. Others, such as stair-descending and walking, can hardly be recognized from the raw signals. Each activity has its own sEMG patterns in the four channels of signals. This issue reflects the difference in signal patterns of four muscles on lower limb.

### 4.1. Class Separability Results

**Figure 4:**
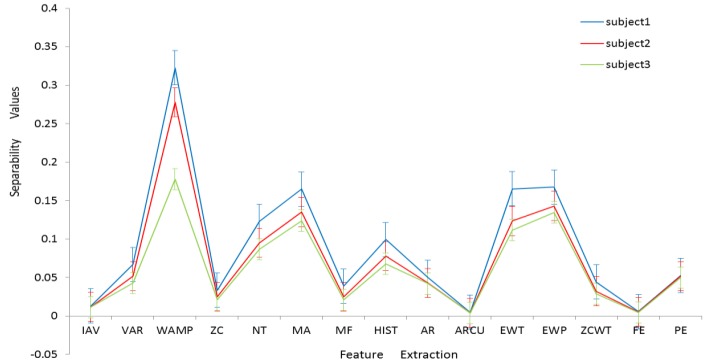
Class separability index values (Error bar: standard error).

Figure 4 illustrates class separability index values (refer to Section 3.2) of the 15 types of EMG feature sets (Table 2 and Section 3.1) for each of three subjects. A high class separability score means that the corresponding feature data are highly separable. The WAMP feature is ranked as the top one, followed by MA, EWT, and EWP. The IAV, ARCU, and FE features are the worst ones.

Figure 4 also shows that there is no significant individual difference in the separability value of EMG features. The average of Spearman’s rank correlation coefficient value between subjects is almost 0.98, indicating that the type ranking hardly vary among individuals. This result indicates that the main results in our study remain intact even for a small number of subjects with a large number of samples for each individual subject. Besides, there is no considerable difference in inherent characteristics of EMG signals between subjects with disabilities and subjects without disabilities [3].

Calculation complexity is an important factor in online applications, particularly in fall detection. The complexity is normally reflected in the calculation time. In the current study, it was calculated on a PC (Intel Core i5-4210U at a 2.4-GHz CPU with a 4G RAM), using MATLAB R2013.

**Figure 5:**
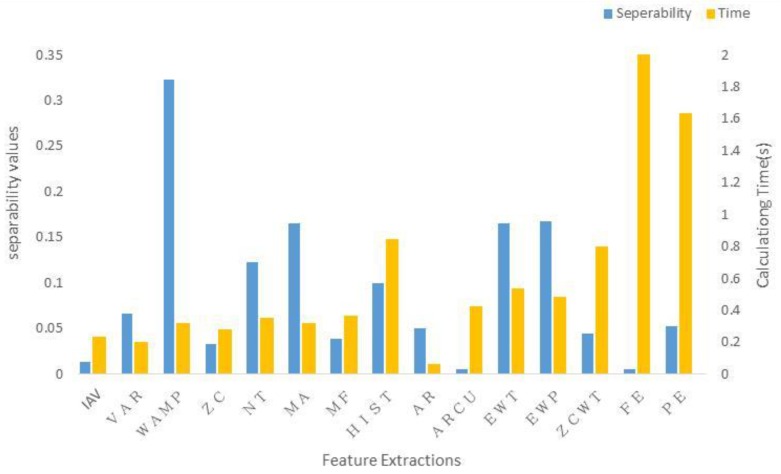
The separability index and calculation time of fifteen features.

**Figure 6:**
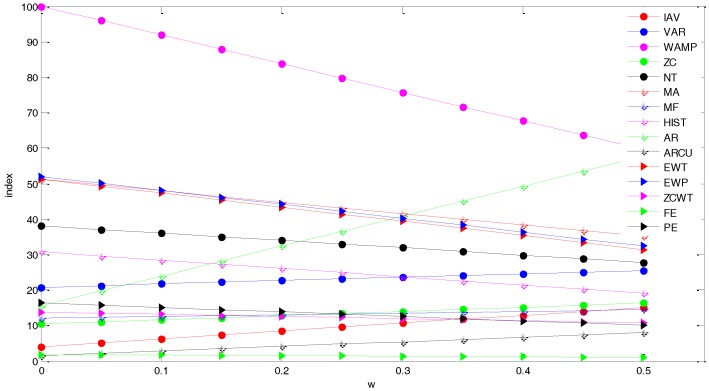
The value of the performance index with various w values.

Figure 5 shows the class separability values and calculation time, which were averaged across subjects, for each individual feature type. The results illustrate that although some feature types have a good separability, some of them like the EWT and EWP, which get better separability values than many other features, have a very long calculation time. Considering this issue, the paper introduced a performance index to trade off the separability value against calculation time, which is defined as:

(16) index=(1−w)×a+w×t

where *a* denotes the normalized separability of each extraction method, *t* means the inverse normalized calculation time, *w* (ranges 0 to 1) denotes the proportion of the computational cost in the algorithm. The fastest and the best separability equalized to 100, and the rest are quantified by their respective proportions. According to this equation, the higher *index* means the better feature. Since the separability always plays a more important role in an arithmetic, the w’s range was selected from 0 to 0.5 and the interval was equalized to 0.05. Figure 6 illustrates that regardless of calculation time (w = 0), all of WAMP, MA, EWT and EWP performed well. As expected, the index of AR was mostly affected by the time. When the calculation time weighs greater than 0.3, AR becomes better than others, except for WAMP. The figure also shows that taking into account the time, the WAMP still ranked first among those feature types.

### 4.2. Activities Recognition Results

**Figure 7:**
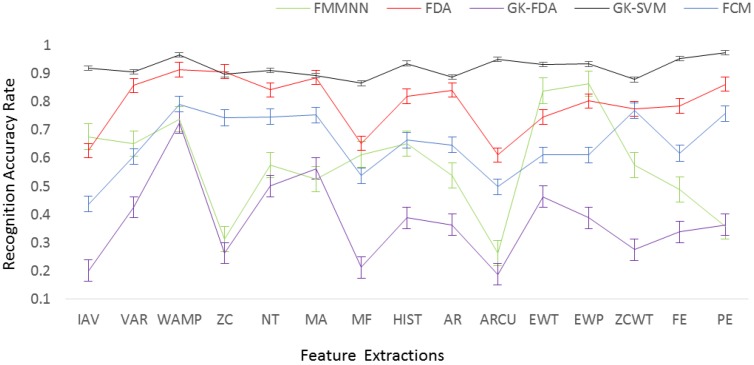
Average of Recognition Accuracy Rates (error bar: standard error).

The feature dataset of seven kinds of ADLs and falls was individually input into five types of classifiers (Table 2). All simulations were performed using a fivefold cross validation. The dataset was divided into five equal-sized subsets. Among those subsets, one of the subsets was chosen as testing data and the remaining subsets as training data. This process was repeated for each subset, resulting in five results. The results averaged over five sub-data sets are showed in Figure 7.

The coefficients in algorithm always play an important role to judge the goodness of an algorithm. Hence, to perform simulations of the FMMNN, the exhaustive search was employed. It means that the best result out of all experiments with a wide range of parameter values was chosen. To perform simulations of the GK-LDA and the GK-SVM, the best result among all experiments with a number of parameter values varying from 0.5 to 5 with a step size of 0.1, was chosen. 

The average of recognition accuracy rates can be seen in Figure 7 and Table 3. The calculation time, which is the time of feature extraction and the time of pattern recognition, is shown in Table 3. The GK-SVM using the PE feature ranked first at 97.35%. The classifier GK-SVM with IAV, MF, AR, FE and PE features delivered the recognition rates of above 95% which are satisfied for activity monitoring. The classifier GK-SVM with all features resulted in the calculation time below 50 ms. 

**Figure 8:**
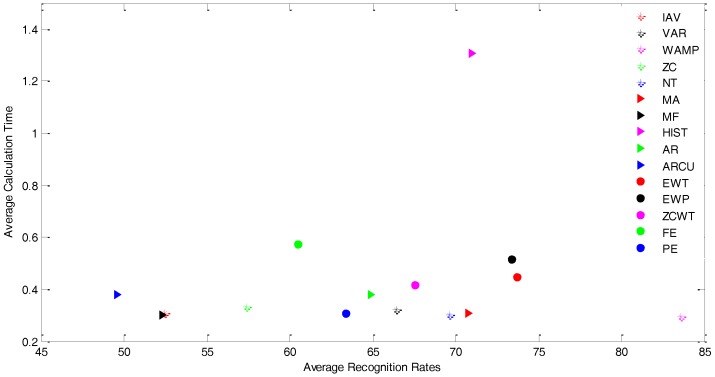
The average recognition accurate rates vs. the average calculation time of 15 features across the classifiers.

The GK-SVM gets the highest recognition rate for all feature types except for the ZC. The GK-SVM gets also the lowest calculation time for all feature types. The best feature is WAMP for all classifiers except for FMMNN, with which the EWP feature is the best feature. Figure 7 also illustrates that the GK-SVM has the minimum variance for all feature types. Figure 8 is a plot of the average recognition accurate rates versus the average calculation time of 15 features across the classifiers. This figure clearly shows that the best features are in the bottom right of the figure.

### 4.3. Fall Detection Results

**Figure 9:**
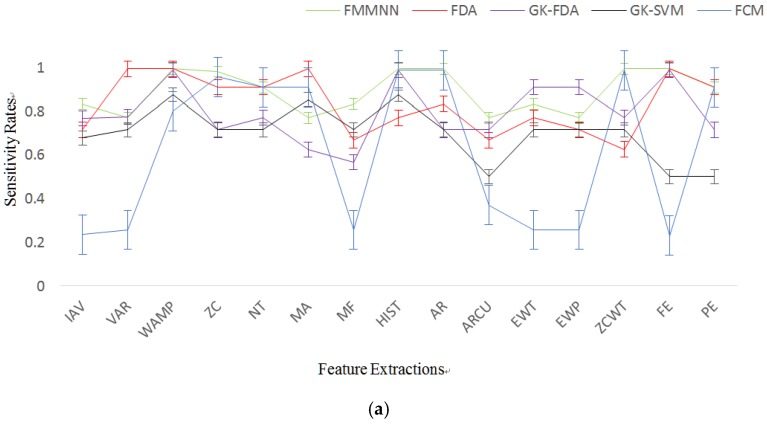

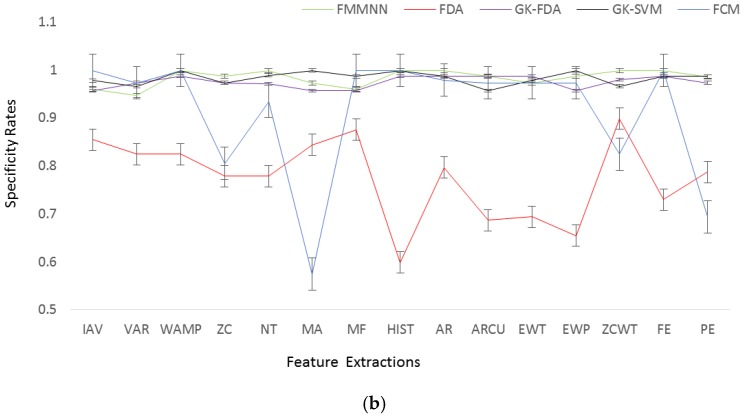
Sensitivity (SEN) and specificity (SPE). (**a**) Average sensitivity (error bar: standard error), (**b**) Average specificity (error bar: standard error).

All seven activities in ADLs are classified as type one and the trip-fall as type two. The used recognition method is the same as that of Section 3.3. Figure 9 and Table 4 show the sensitivity (SEN, falls identified correctly), the specificity (SPE, ADLs identified correctly), and the calculation time. The highest sensitivity is 99.35% that belongs to two classifiers. The first classifier is the FMMNN with the WAMP, HIST, AR, ZCWT, and FE features and the second classifier is the FDA with the VAR, WAMP, MA, and FE features. All classifiers with all feature types have good specificity of above 95%, except the FDA with all feature types and the FCM with the feature of ZC, MA, ZCWT and PE. However, the FCM is the worst in terms of both the sensitivity and specificity. Besides, the performance of LDA was poor in the specificity. It is worth noting that the false positive was mainly caused by the stand-to-sit (Channel 1 of the Figure 3), whose signal is similar to trip-falls. Although the FMMNN classifier regardless of feature types has the best performance in both of sensitivity and specificity, its calculation time is longest. The classifier GK-FDA with the feature WAMP delivered high sensitivity (98.70%) and specificity (98.59%) and a short calculation time (65.586 ms), which is satisfied for pre-impact fall detection.

**Figure 10:**
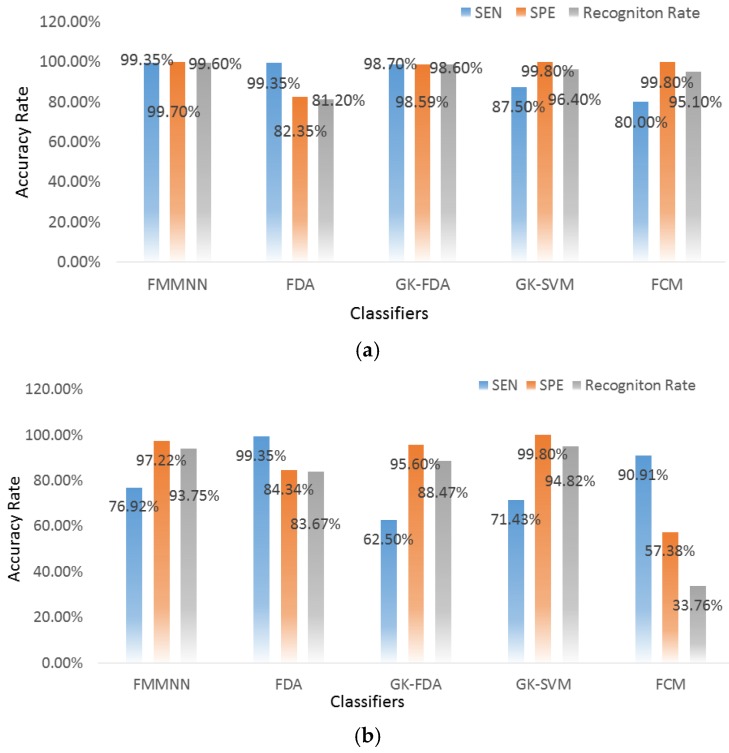
Sensitivity, Specificity, and recognition accurate rate of two specific feature types. (**a**) Sensitivity, Specificity, and whole recognition rate of WAMP. (**b**) Sensitivity, Specificity, and Recognition Rate of MA.

The WAMP and MA, which are two feature types with high recognition rates in ADLs-recognition, were chosen and their sensitivity, specificity, the total accuracy recognition rates were analyzed. The results are shown in Figure 10. The WAMP, FMMNN, and GK-FDA features performed well in all three rates. The GK-SVM has a high specificity but its sensitivity drops to 87.5%, meaning that it cannot recognize tip-falls perfectly. Although the GK-SVM for the MA feature has a specificity of 90%, but its sensitivity is even lower than the others’. It indicates that the GK-SVM method is not an appropriate choice for this process. 

## 5. Discussion

The purpose of this study was to find an optimal combination of sEMG feature types and classification methods, thereby providing a practical guideline for designing a sEMG based activity monitoring and fall detection system. The results of this study demonstrate that a system with four sEMG sensors was sufficient for achieving the sensitivity and specificity results in the 90% range, with less than 10% misclassifications. This study provides evidence that automated monitoring of a variety of activities of daily living and fall detection can be achieved using a wireless and wearable surface EMG sensor system with feature extraction and pattern recognition techniques. 

There are several basic limitations associated with this study that need further development to provide a wearable sEMG-based activity monitoring and fall detection system for the elderly or patients that can be used under real-world conditions. The authors of the current research study monitored “scripted” daily activities and simulated trip falls performed by healthy volunteers in a laboratory environment. It is not known how well this algorithm would work in a real scenario with unscripted free-form activities performed by elderly or real patients. In this study, each individual was trained separately and required multiple repetitions of the task to obtain sufficient data for training and testing purposes. However, it is not clear how different it is from identifying activities and falls in real life with a larger task set. These conditions need to be investigated before using these algorithms for clinical purposes.

## 6. Conclusions

Based on the accuracy of recognition rate and computational complexity, a series of methods of surface EMG feature extraction and recognition were estimated for activity monitoring and fall detection. The statistical analysis of fifteen types of EMG feature sets determined that the WAMP, MA, EWT, and EWP features are highly separable and the IAV, VAR, and AR features have the shortest calculation time. The statistical analysis of class separability against calculation time recognized the WAMP, AR, and MA as the most advantageous features. In terms of activity monitoring, the WAMP is the best feature, the GK-SVM is the best classifier, and the combination of the GK-SVM and PE is the best possible combination of EMG feature types and classification methods. In terms of fall detection, the FMMNN classifier has the best performance in the sensitivity and specificity, but the longest calculation time. Since the detection time for realizing pre-impact fall detection must be less than 300 ms [1], the best choice is the GK-FDA classifier with the WAMP feature whose sensitivity and specificity are both above 98% and the calculation time is 65 ms. 

This system would further reduce recognition errors if combined with mechanical sensors such as accelerometer or gyroscope sensors. This idea helps to achieve both high recognition rate and reliability for the development of activity monitoring and fall detection systems. Besides, it also has important implications for other EMG signal-based devices, such as clinical assistive devices, walking assist devices, and robotics or prosthetic devices.

## Figures and Tables

**Table 1:** List of features.

ID	Extraction Feature	Acronym	Dimension
1	Integral of Absolute Value	IAV	4
2	Variance	VAR	4
3	Wilson Amplitude	WAMP	4
4	Zero Crossing	ZC	4
5	Number of Turns	NT	4
6	Mean of Amplitude	MA	4
7	Mean Frequency	MF	4
8	Histogram	HIST	84
9	Auto-Regressive Coefficient	AR	12
10	Auto-Regressive Coefficient From Third-Order Cumulant	ARCU	12
11	Energy of Wavelet Coefficient	EWT	20
12	Energy of Wavelet Packet Coefficient	EWP	32
13	Zero Crossing of Wavelet Coefficient	ZCWT	20
14	Fuzzy Entropy	FE	4
15	Permutation Entropy	PE	4

**Table 2:** List of classification methods.

Classification Algorithm	Acronym
Fisher Discriminant Analysis	FDA
Fuzzy Min-Max Neural Network	FMMNN
Kernel Linear Discriminant Analysis	GK-FDA
Kernel Support Vector Machine	GK-SVM
Fuzzy C-Means	FCM

**Table 3:** Recognition Rates and Calculation Time (%, ms). The bold numbers indicate the features with the highest accuracy rate for each classifier. The shaded numbers indicate the classifiers with the highest accuracy rate for each feature. The zigzag underlines indicate the features with shortest calculation time for each classifier. The straight underlines indicate the classifiers with shortest calculation time for each feature.

	FMMNN	FDA	GK-FDA	GK-SVM	FCM
IAV	67.50 800.94	62.60 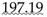	20.00 77.563	91.73 49.61	43.59 402.28
VAR	65.00 789.70	85.66 284.16	42.50 59.978	90.41 40.54	60.47 427.47
WAMP	73.75 771.37	**91.24** 210.39	**72.50** 65.17	96.43 46.36	**79.06** 356.37
ZC	31.25 865.25	90.60 266.96	26.25 64.248	89.59 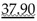	74.22 409.71
NT	57.50 867.31	84.10 197.22	50.00 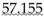	91.02 48.58	74.53 309.41
MA	52.50 807.75	88.42 301.44	56.25 66.18	89.18 39.04	75.23 297.96
MF	61.25 796.68	65.10 218.02	21.25 70.932	86.43 49.71	53.75 356.09
HIST	65.00 4654.2	81.80 881.89	38.75 78.112	93.47 47.23	66.33 825.38
AR	53.75 1204.1	83.96 285.61	36.25 71.317	88.60 49.21	64.61 282.48
ARCU	26.25 1177.9	61.05 256.22	18.75 57.929	95.00 49.28	49.77 322.36
EWT	83.75 1322.6	74.49 336.01	46.25 64.091	93.06 45.35	61.09 417.13
EWP	**86.25** 1671.0	80.17 336.97	38.75 58.699	93.27 43.54	61.02 430.19
ZCWT	57.50 1236.7	77.36 281.92	27.50 57.94	87.76 39.83	76.80 398.21
FE	48.75 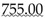	78.42 214.81	33.75 76.945	95.20 56.50	61.64 261.77
PE	35.75 793.57	86.11 278.59	36.25 70.546	97.3597.35 50.31	75.70 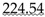

**Table 4:** Sensitivity, Specificity and Calculation times (%, % and ms). The bold numbers indicate the features with highest Sensitivity and Specificity rate for each classifier. The shaded indicate the classifiers with the highest Sensitivity and Specificity rate for each feature. The zigzag underlines indicate the features with shortest calculation time for each classifier. The straight underlines indicate the classifiers with shortest calculation time for each feature.

	FMMNN	FDA	GK-FDA	GK-SVM	FCM
IAV	83.33 95.89 798.171	71.43 85.37 22.238	76.60 95.60 71.658	67.70 97.70 31.680	23.53 99.80 80.084
VAR	76.92 74.60 774.504	99.35 82.35 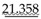	77.40 97.22 67.319	71.43 96.50 26.893	25.64 97.22 63.271
WAMP	99.3599.35 **99.70** 914.863	99.35 82.35 21.554	98.70 98.59 65.586	**87.50** 99.8099.80 28.058	80.00 99.80 66.866
ZC	98.10 98.59 985.637	90.91 77.78 23.987	71.43 97.22 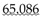	71.43 97.22 29.470	95.60 80.46 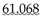
NT	90.91 99.7099.70 782.313	90.91 77.78 24.333	76.92 97.00 82.546	71.43 98.80 30.584	90.91 93.33 68.891
MA	76.92 97.22 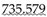	99.3599.35 84.34 24.583	62.50 95.60 72.768	71.43 99.8 26.443	90.91 57.38 76.955
MF	83.33 95.89 785.180	66.67 87.50 22.957	56.60 95.60 86.225	71.43 98.59 31.620	25.64 99.80 65.238
HIST	**99.35** **99.70** 1768.134	76.92 59.83 36.520	98.7098.70 **98.59** 82.650	71.43 99.8 35.755	98.7098.70 99.8099.80 106.047
AR	99.3599.35 99.7099.70 871.808	83.33 79.55 22.326	71.43 98.59 84.999	71.43 98.59 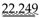	98.70 97.80 68.527
ARCU	76.92 98.59 916.730	66.67 68.68 29.769	71.43 98.59 67.665	50.00 95.60 38.354	37.04 97.22 66.568
EWT	83.33 97.22 1089.698	76.92 69.31 32.745	90.91 98.59 95.472	71.43 97.70 34.491	25.64 97.22 70.804
EWP	76.92 98.59 1343.922	71.43 65.42 32.826	90.91 95.60 88.787	71.43 99.80 38.128	25.64 97.22 121.709
ZCWT	99.3599.35 99.7099.70 1010.854	62.50 **89.74** 45.618	76.92 97.90 81.515	71.43 96.50 45.383	98.7 82.35 73.311
FE	99.3599.35 **99.70** 747.180	99.35 72.92 398.935	98.70 98.59 437.390	50.00 98.59 398.873	22.99 99.80 432.002
PE	90.91 98.50 820.835	90.91 78.65 46.068	71.43 97.22 86.390	50.00 98.59 50.373	90.91 69.31 86.616
